# Evaluation of community-based HIV self-testing delivery strategies on reducing undiagnosed HIV infection, and improving linkage to prevention and treatment services, among men who have sex with men in Kenya: a programme science study protocol

**DOI:** 10.1186/s12889-019-7291-2

**Published:** 2019-07-23

**Authors:** Parinita Bhattacharjee, Dorothy Rego, Helgar Musyoki, Marissa Becker, Michael Pickles, Shajy Isac, Robert Lorway, Janet Musimbi, Jeffrey Walimbwa, Kennedy Olango, Samuel Kuria, Martin Kyana Ongaro, Amy Sahai, Mary Mugambi, Faran Emmanuel, Sharmistha Mishra, Kigen Bartilol, Stephen Moses, James Blanchard

**Affiliations:** 1 Centre for Global Public Health, University of Manitoba, Winnipeg, Canada; 2 Partners for Health and Development in Africa, University of Manitoba, Geomaps Building, Upper Hill, Nairobi, Kenya; 3 Nutrition International, Ottawa, Canada; 4 National AIDS and STI Control Programme, Ministry of Health, Nairobi, Kenya; 5 Imperial College, London, UK; 6 G10 Research Advisory Committee, Nairobi, Kenya; 7 Men Against AIDS Youth Group, Kisumu, Kenya; 8 Mamboleo Peer Empowerment Group, Kiambu, Kenya; 9 HIV and AIDS People’s Alliance of Kenya, Mombasa, Kenya; 10 India Health Action Trust, New Delhi, India; 11 Department of Medicine, St. Michael’s Hospital, University of Toronto, Toronto, Canada; 12 Institute of Medical Sciences, University of Toronto, Toronto, Canada; 13 Institute of Health Policy Management and Evaluation, Dalla Lana School of Public Health, University of Toronto, Toronto, Canada

**Keywords:** MSM, HIV self testing, HIV prevention, Evaluation, Kenya

## Abstract

**Background:**

HIV prevalence among men having sex with men (MSM) in Kenya is 18.2%. Despite scale-up of HIV testing services, many MSM remain unaware of their HIV status and thus do not benefit from accessing HIV treatment or prevention services. HIV self-testing (HIVST) may help address this gap. However, evidence is limited on how, when, and in what contexts the delivery of HIVST to MSM could increase awareness of HIV status and lead to early linkage to HIV treatment and prevention.

**Methods:**

The study will be embedded within existing MSM-focused community-based HIV prevention and treatment programmes in 3 counties in Kenya (Kisumu, Mombasa, Kiambu). The study is designed to assess three HIV testing outcomes among MSM, namely a) coverage b) frequency of testing and c) early uptake of testing. The study will adopt a mixed methods programme science approach to the implementation and evaluation of HIVST strategies via: (i) a baseline and endline bio-behavioural survey with 1400 MSM; (ii) a socio-sexual network study with 351 MSM; (iii) a longitudinal qualitative cohort study with 72 MSM; (iv) routine programme monitoring in three sites; (v) a programme-specific costing exercise; and (vi) mathematical modelling. This protocol evaluates the impact of community-based implementation of HIV self-testing delivery strategies among MSM in Kenya on reducing the undiagnosed MSM population, and time for linkage to prevention, treatment and care following HIV self-testing. Baseline data collection started in April 2019 and the endline data collection will start in July 2020.

**Discussion:**

This study is one of the first programme science studies in Sub-Saharan Africa exploring the effectiveness of integrating HIVST interventions within already existing HIV prevention and treatment programmes for MSM in Kenya at scale. Findings from this study will inform national best approaches to scale up HIVST among MSM in Kenya.

## Background

Kenya has the joint fourth largest HIV epidemic in the world, alongside Mozambique and Uganda, with the national adult HIV prevalence estimated at 4.9% in 2017 [1]. A disproportionate number of new infections occur in specific populations in Kenya, which include key populations (KPs) [2, 3]. KPs in Kenya include female sex workers (FSWs), men who have sex with men (MSM) and people who inject drugs (PWID) and are prioritized in the national HIV response [4]. Although sex work, same sex relationships and drug use are criminalized in Kenya, the Ministry of Health has scaled up HIV prevention and treatment programmes among KPs in 34 out of 47 counties [5].

Despite the progress made in HIV prevention among KPs globally, MSM continue to experience a high burden of HIV infections compared to the general population [6]. The risk of acquiring HIV is 27 times higher among MSM compared to other heterosexual men [7]. Early contact with prevention and treatment programmes for KPs can be effective in reducing HIV transmission and mortality among those who are HIV positive [8]. As HIV testing is the entry point for HIV prevention and treatment [9, 10], new and better testing approaches to reach individuals who are undiagnosed are needed [11]. Prioritizing early HIV diagnosis [4] has led to the expansion of HIV testing services in Kenya, in both facility and community settings [12].

Even with the expansion of testing services, the Kenya KP National Programme data show that only 53% of the estimated MSM living with HIV were known and registered in KP programmes in December 2018 [13]. Despite improvements in policies and access to HIV testing services, MSM in Africa and Kenya still experience a number of barriers including stigma, difficulties in disclosing their sexual orientation, confidentiality concerns, access to services, inadequate support services, and fear of blood/finger prick testing [14, 15]. HIV self-testing (HIVST) provides a promising approach to promote HIV testing among MSM [16]. A systematic review and meta-analysis exploring the effect of HIVST on HIV testing behaviours, showed that HIVST could increase HIV testing frequency among MSM and would reach those MSM who are first-time testers or who test infrequently such as those who are married or suspect that they are HIV-positive [17]. The analysis included evidence from resource-limited country settings, but there was no evidence included from Africa [17].

Very few studies have examined any aspect of HIVST among MSM in Sub-Saharan Africa [18–20]. Kenya has conducted several studies to understand the feasibility of scaling up HIVST within the general population, including among sero-discordant couples and FSWs, yet there is little information on HIVST among MSM specifically [21–24]. Although global evidence suggests that introduction of HIVST can benefit MSM, evidence is limited on how, when, and in what contexts the delivery of HIVST to MSM could increase awareness of HIV status, and lead to early linkage to HIV treatment and prevention services in the African context. Therefore, key programmatic questions remain on how to effectively scale up the delivery of HIVST for MSM, and to support those persons that test positive with treatment, and those that test negative with access to prevention programmes.

The University of Manitoba, in partnership with the National AIDS and STI Control Programme (NASCOP), G10 (a MSM research network in Kenya), and three MSM led community-based organizations (CBOs) in Kenya (Mamboleo Peer Empowerment Group (MPEG) in Kiambu, Men Against AIDS Youth Group (MAAYGO), and the HIV & AIDS People’s Alliance of Kenya (HAPA Kenya) in Mombasa), is conducting a study to evaluate the impact of community-based implementation of HIVST delivery strategies among MSM in Kenya. This proposed evaluation study will examine how HIVST can reduce the overall size of the undiagnosed MSM population and affect linkage to prevention and treatment in those who self-test. Lessons learned from this study will inform policy and practices nationally, and is well timed as Kenya examines implementation and scale up of HIVST.

## Methods/design

### Study settings

This study will be embedded in existing community-based HIV prevention and treatment programmes among MSM in 3 counties in Kenya (Kisumu, Mombasa, Kiambu). The population estimates of these counties ranges from 0.9 to 1.6 million, with Kiambu being the most densely populated. Collectively there are 6 partners that implement HIV prevention and treatment programmes in these counties. The study will partner with the MSM-led CBOs in each study site which are currently implementing HIV prevention and care programmes for MSM in these counties.

The overall HIV prevalence in the general population in Kisumu, Mombasa, and Kiambu is 16, 4, and 4%, respectively [1]. In 2017, the self-reported HIV prevalence among MSM was 13, 19 and 23% in Kisumu, Mombasa and Kiambu respectively [25]. Recent size estimation studies of hotspots (physical spaces where MSM meet other sexual partners) estimate that there are 2,855, 2,492 and 1,664 MSM in Mombasa, Kisumu and Kiambu counties respectively [26]. However, internet-based mapping conducted by NASCOP, in partnership with the University of Manitoba and the CBOs, indicate that approximately 25% of MSM seeking sexual partners over the internet do not visit hotspots [27]. This suggests that the estimates of MSM in the counties could be higher than the hotspot-based estimates. There is little data on the uptake and frequency of testing of MSM that do not visit hotspots, but a key part of the intervention will be to try to ensure that this group is reached.

### HIVST intervention

The intervention design and implementation will be informed by National Guidelines for HIV and STI Programme for Key Populations [28], the national implementation guidelines for HIV and STI programming among young KPs [29], and HIVST operational guidelines [30]. The HIVST intervention will be embedded within already existing HIV and sexual health programmes implemented by the three partner CBOs in their respective counties. The interventions will target all MSM above the age of 15 years in each county. The HIVST intervention will use different service delivery mechanisms to make HIVST accessible to MSM in the counties, including distribution through facility and community settings. Facility distribution will include: (i) clinics and Drop-in Centres (DICs) operated by the KP programmes; and (ii) outreach clinics conducted in the hotspots by the KP programmes. Community distribution will include: (i) direct distribution through MSM peer educators at hotspots and where MSM usually meet, such as parties, clubs, and DIC events; and (ii) indirect distribution through the social and sexual networks of the MSM who are known to the programme. Indirect distribution will focus on reaching those who are not enrolled and reached by the programmes. The HIVST kits will be distributed by trained personnel that will include clinicians, nurses, outreach workers, peer educators and peers. Along with the kits, the personnel will provide information and education about sexuality, risks of unsafe sexual behaviours, HIV testing services, and prevention and treatment services, in a targeted way to specifically reach those who are normally unreached by programmes. The focus of the peer and clinical interaction will be on building confidence and skills to use HIV test kits and seeking prevention and treatment services after testing. MSM will have the option to choose either assisted (supported and in presence of outreach or clinical staff) or unassisted (on their own) self-testing, depending on their preference. Demand generation for HIVST will be conducted at the facility during hotspot-based peer contacts, DIC events, special events established to promote HIVST, and through virtual media such as Facebook and WhatsApp groups, which are used to find sexual partners. Linkage to prevention and treatment services following HIVST is essential. Hence, referral cards and information will be provided along with the test kits during distribution. Follow-up to the MSM who received HIVST directly will be done by trained personnel. Text messages and calls will be made to the MSM who receive HIVST directly to remind them about follow-up with prevention and treatment services. The Ministry of Health HIV testing website (https://www.besure.co.ke/) will be also advertised so that MSM who do not want to contact the programme can seek services through the website. The National HIV Testing Helpline, 1190 [31], will also be promoted. The intervention will be implemented for 12 months and hopes to reach 3,000 MSM who are either non-testers or infrequent testers.

### Programme science framework

A defining feature of this evaluation study is its use of a Programme Science approach. The Programme Science approach systematically applies scientific theory and empirical knowledge to answer critical programmatic questions [32]. At the core of programme science is the systematic combination of the programme cycle with a research strategy, by embedding research within programmes and having programmes set and drive the research agenda [33]. In the context of this research, the key question is derived from the challenges that HIV programmes with MSM are experiencing of low uptake and low frequency of HIV testing among MSM in Kenya. This research project will encompass the programme science framework by ensuring that scientific enquiry is driven by three core spheres, namely: a) strategic planning: collecting necessary contextual and population-based evidence to understand current unmet gaps in testing, the needs of the population, and preferable service delivery options for HIVST to prioritise programme design; b) programme implementation: implementation of the designed programme, which includes a combination of testing options and delivery mechanisms through integration within existing HIV prevention and treatment programmes for MSM; and c) programme evaluation: monitoring and assessment of the impact of the programmes using a range of methods to make space for responsive tactical and strategic adaptation of programme strategies. A Programme Science approach also encourages participatory engagement with affected communities [26, 34]. This study is being conducted in partnership with community-based organizations and the Ministry of Health in Kenya.

### Evaluation design

#### Hypothesis

**Fig. 1:**
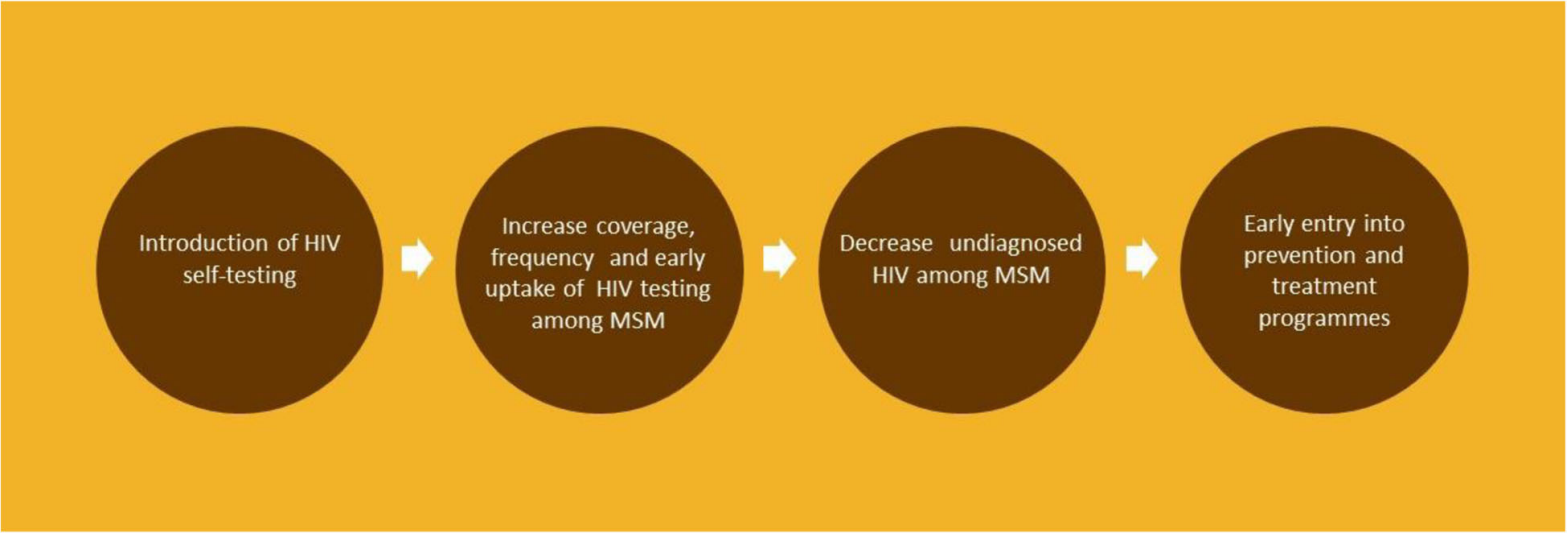
Hypothesis of the study

Our central hypothesis (Fig. 1) is that the introduction of HIVST will lead to fewer undiagnosed MSM in Kenya by increasing coverage, frequency, and early uptake of HIV testing among MSM, which in turn will lead to a decrease in undiagnosed HIV among MSM, and early entry into HIV prevention and treatment programmes.

#### Evaluation questions

The key evaluation questions can be categorized under four domains; (1) HIV testing; (2) linkage to treatment and prevention; (3) delivery mechanisms; and (4) impact on population. A summary of evaluation study questions is listed in Table 1.

**Table 1:** Evaluation domains and questions

Domains	Evaluation questions
HIV testing	To what extent will HIVST improve the coverage, frequency and early uptake of HIV testing?
Linkage to treatment and prevention	Does HIVST reduce or increase access to linkage to treatment among those with an HIV positive test, compared to other testing methods? Does HIVST reduce or increase access to linkage to prevention programmes among those with a HIV negative test result, compared to other testing methods?
Delivery mechanisms	Which HIVST delivery mechanisms will be most effective in improving uptake and frequency of HIV testing and linkage to treatment and prevention? What will be the estimated costs of different service delivery mechanisms?
Impact	To what extent would HIVST programme interventions reduce the overall size of the undiagnosed MSM population? To what extent would HIVST programme interventions reduce the transmission of HIV in the context of MSM populations and networks?

#### Study outcomes

The primary outcomes will assess the impact of the intervention on.

1. increasing coverage of HIV testing among MSM.
2. increasing frequency of HIV testing among MSM.
3. increasing early uptake of HIV testing among MSM.

The study will also assess the following secondary outcomes:

4) effectiveness of different delivery mechanisms on improving the coverage and frequency of HIV testing in different sub populations of MSM.
5) effectiveness of linkage to HIV prevention and treatment services for MSM who self-test.
6) cost effectiveness of different HIVST service delivery mechanisms.
7) population-level impact of the introduction of HIVST in reducing the size of the undiagnosed MSM population and on HIV transmission.

#### Study time line

The study will be completed in 2 years. The baseline data collection will take place during April – June 2019 and the endline data collection is expected between July – September 2020. Data cleaning and analysis is expected to take 4–5 months after endline data collection. Dissemination of the results will be done by March 2021.

#### Evaluation methods

The evaluation will employ a mixed method design that involves analysis of primary data (both qualitative and quantitative data) and additional analyses of secondary data (routinely collected aggregated and anonymized programme monitoring data). The overall evaluation methods include the following and are described in further detail below:

1. baseline and endline bio-behavioural survey
2. sexual and social network study
3. longitudinal qualitative cohort study
4. routine programme monitoring
5. programme specific costing exercise
6. mathematical modelling

##### Baseline and Endline bio-Behavioural survey

The baseline and endline bio-behavioural survey will address the following primary outcomes 1–3 and secondary outcomes 4–5.

#### Inclusion criteria

The inclusion criteria for the bio–behavioural survey will include:

- identify as male.
- engaged in anal or oral sexual intercourse with another male in the previous 12 months.
- age 15 years and above. This inclusion criterion aligns with the national HTS guidelines, which stipulate the minimum age for HIV testing without parental/ guardian consent as 15 years [12].

#### Sample size

Sample sizes for the quantitative baseline and endline surveys have been calculated based on assumptions in which baseline prevalence and expected change in prevalence were varied to arrive at a minimum sample size. Behavioural data from previous surveillance, as well as routine programme data, were reviewed to determine baseline prevalence estimates to inform the sample size calculation.

To calculate the sample size for this study, the basic parameter considered is the level of change in the rates of HIV testing, i.e. among those who are HIV positive, how many know their status, and what is the net change between various survey rounds. Thus, the basic parameter used for the sample size calculation is the estimated change in prevalence of known HIV status among those MSM who are HIV-positive in the three counties.

We wanted to have enough power (90%) to detect the difference between P1 and P2 (knowledge of HIV status at baseline and endline) with precision. We assumed a relative change of 10–15% in the percentage of HIV positive MSM who know their status between P1 and P2, and used the following formula to determine sample size:

$$ n=D\frac{{\left[\sqrt{2P\left(1-P\right){Z}_{1-a}}+\sqrt{P_1}\left(1-{P}_1\right)+{P}_2\left(1-{P}_2\right){Z}_{1-\beta}\right]}^2}{\Delta^2} $$

P1 = estimated prevalence of known HIV status among those HIV-positive at baseline.

P2 = expected prevalence of known HIV status among those HIV-positive in future (detect a change of 10–15%).

P = (P1 + P2) / 2.

∆^2^ = (P2 – P1)^2^.

Z_1-α_ = 95% level of significance.

Z_1-β_ = Power of the study, set at 90%.

D = Design effect of the study, assumed to be 2.

Based on these assumptions, a minimum sample size of 1,140 was calculated. The sample size was further increased by 5% to account for non-response, to arrive at a national sample size of 1200 for the study, or 400 in each site/county.

#### Sample selection and recruitment

The required sample will be distributed equally to each county i.e., 400 for each site. This sample size provides a reasonable power (~ 80%) at the county level to estimate a 10–15% change in the prevalence of known HIV status between baseline and endline.

MSM meet their sexual and social partners in both geographical hotspots and virtual spots (dating apps/Facebook, etc.). Hence, further allocation of the sample within the categories of hotspot-based and virtual site-based MSM will be in equal proportions. Thus, a sample of 200 hotspot-based MSM and 200 virtual site-based MSM will be sampled within each county, using a multi-stage cluster sampling technique, as follows:

### Hotspot based MSM sampling.

Two stage cluster sampling method will be used to sample for hotspot-based MSM. Within each county we propose to include and represent all sub-counties where the MSM CBOs are working. Therefore, the hotspot-based MSM sample (200 MSM) will be distributed into different sub-counties in proportion to the estimated size of MSM using those hotspots.

In the first stage, hotspots will be selected from the available spot lists within each sub-county. This list is available from a recent key population size estimate exercise conducted by NASCOP in 2018–19 [29]. Selection of a spot will be done using a computerized random list. The recruitment strategy is to select two MSM from each spot, who will each then select one additional MSM each from their network. Therefore, the number of spots to be selected in each sub-county will be the total sample in each sub-county divided by 4.

Field research teams (4 members in each site) will visit randomly selected spots and, with the support of peer educators in the project, will randomly select two MSM to participate in the study. The selected MSM from the spot will be screened and, if eligible, will be requested to participate in the study. If the potential participant consents, then a face-to-face interview will be conducted by the researcher. At the end of the interview, the respondents will be requested to make a list of all MSM contacts (the respondent can provide the last 4 digits of their phone number or nick names). The researcher will then randomly select one MSM from the list and would ask the respondent to speak to the selected MSM from the list for consent to participate in the interview. If the selected MSM consents for a face-to-face interview by a researcher, then the researcher will contact him and set up a time and place for a face-to-face interview. If the selected MSM disagrees, then another name will be picked from the list and the same process will continue. Hence every MSM randomly selected in the hotspot will introduce the researcher to another MSM from his social and sexual network for the interview.

### Virtual site-based MSM sampling.

A multistage sampling approach will also be used for this segment of the MSM population. At the first stage, websites and digital apps used in the counties will be listed by the researchers, along with an estimation of MSM active on these sites. The researchers will take help from the CBOs and MSM staff in the CBOs to develop this list. Each of the sites/apps will be allotted a weight derived from the number of MSM estimated at each virtual site (including Facebook, WhatsApp groups, various MSM networking apps, etc.). The required sample will be allocated to various virtual sites in proportion to the estimated size of these sites.

The next stage will involve random recruitment of MSM operating through these virtual sites. Peer members will log into the various virtual sites at random timings, randomly select the required number of individuals from the ones online at the time of login, and invite them to participate in the survey. Although high non-response may be an issue, the goal is to achieve a representative sample of the MSM community operating through websites and digital apps. The selected MSM will be screened and, if eligible, will be requested to participate in the study. If the MSM consents, then a face-to-face interview will be conducted by the researcher at a time and space convenient to the respondent. At the end of the interview, the respondents will be requested to make a list of all his MSM contacts similar to the process conducted in the hotspot-based sampling. The researcher will then randomly select one MSM from the list, explain the study, and ask the respondent to enquire about the individual’s interest to participate. If interested, the interviewer will call the individual and invite him to attend a face-to-face interview at a time and place convenient to the participant. Hence, every MSM randomly selected in the virtual sites will introduce the researcher to another MSM from his social and sexual network for the interview.

#### Study procedures

All selected study participants will be administered a questionnaire face-to-face by a trained researcher in Kiswahili or English, as preferred by the respondent. Prior to implementing the study, researcher training will also entail a question-by-question discussion and consensus-building process on how to ask each question, based on intent and current terms in common usage. The data will be collected using paper-based questionnaires. All data collection will be conducted in a private room at a time and location that is convenient to the participant. It is anticipated that the majority of people living in the study sites will speak English or Kiswahili. All interviewers will be fluent in English and Kiswahili for administration of consent and the questionnaire. Both the consent form and the questionnaire will be translated by an experienced translator. Eligible potential participants will be able to read, or have read to them, an information sheet for informed written consent, according to their preference, with the opportunity to have any questions answered by the interviewer. The information sheet for consent will cover all procedures, potential risks, benefits, and who to contact in study sites to report complaints or concerns. The document will allow for separate consent for individual components of the survey, including: completion of the questionnaire; provision of a biological sample; and rapid HIV testing with return of results. After the participants clearly understand the contents of the consent form, they will be asked to provide written consent. The proposed study will use non-identifying unique study ID codes for all data components (questionnaire, laboratory, cohort) pertaining to the study. ID codes for all data components of a single individual will be linked. After administering the questionnaire, consenting participants will undergo HIV testing and counselling by an HIV Testing Service (HTS) counsellor who will be a member of the study team.

#### Specimen collection

The HTS counsellor in the study team will provide pre- and post-test counselling and will collect blood samples from consenting participants at the survey site. Rapid testing will be conducted using a serial testing scheme based on the national algorithm for approved test kits. The kits will collect capillary blood from a finger stick using a lancet and capillary tube. All participants who consent will be tested using Alere Determine™ HIV-1/2 Combo (Alere, Florida, United States). Non-reactive results will be considered negative, and reactive results will be confirmed using the First Response Kit (Murex HIV1–2-O, Abbot/Murex, Germany). If the confirmatory test results are nonreactive, results will be recorded as inconclusive. A client with an inconclusive result will be referred to the nearest comprehensive care clinic. A dried blood spot (DBS) sample will also be collected from all participants who provide consent. Blood for the DBS will be taken from a middle finger prick using a lancet and capillary tube. After the interview and sample collection, the participant will be referred to the MSM programme facility, either through accompanied referral by a peer educator or through delivery of a referral card. Counselling will be provided to educate the respondents on the use of HIV self-test kits at the facility as per national guidelines. The DBS samples from the three sites will be transported to the HIV National Laboratory in Nairobi for analysis.

##### 2. Socio–sexual network study

This network study will address the secondary outcome 4. This assessment will help to: a) characterize and identity patterns of connection between different types of service using (or avoiding) by MSM in each site; and b) develop a sampling frame for the longitudinal qualitative study.

#### Inclusion criteria

The inclusion criteria for the study will include:

- age 18 years and above.
- identify as male.
- engaged in anal or oral sexual intercourse with another male in the previous 12 months.

#### Sample recruitment and data collection

All stages of the assessment will be initiated by 12 community researchers (CRs) (4 in each site) who will receive training in research ethics and data collection. The CRs in each site will select a total of 9 seed respondents. Consent will be obtained for participation in the assessment. The seeds will be purposefully recruited from the following type of MSM respondents: 3 MSM who are regular service users (i.e. those who are registered in the CBO programmes in the 3 sites); 3 young MSM (18–24 years) who are not registered in the CBO programmes and have not accessed services in the CBO programme; and 3 older MSM (25 years and above) who are not registered in the CBO programme and have not accessed services in the CBO programme.

All 9 seeds in each site will complete a respondent demographic form. Then using their cellphones, the seeds will be asked to list out the 30–40 contacts (nickname and last 4 digits of their cell phone number) in their social and sexual network using the following criteria: a) MSM in their sexual network with whom they had sex in the last 12 months; b) MSM in their social network with whom they had social contact in the last one-month; and c) female partners in their sexual network with whom they have had sex in the last 12 months. The last 4 digits of their cell phone number will provide a unique code to the contacts listed by the seed. The seeds will fill a short survey for each of their listed contacts. The short surveys will attempt to gather information on: a) age and social economic status; b) location where respondent met and/or had sex; c) whether the person is “out” or “closeted” in their community (i.e. disclosed only to family or friends, or CBO-based health care providers); d) means of connecting with other MSM-MSW (i.e. social media, cruising/hotspots, CBO events); and, if possible, e) engagement with the government KP programme (note: the respondent may need to verify this by telephone if they are uncertain). This exercise will provide a larger sampling frame for the qualitative study with particular inclusion of MSM who are non-programme users. After completing the respondent demographic form and a short survey regarding their sexual and social networks, each primary seed will recruit three MSM respondents from their contact list using the following criteria: a) one MSM who is registered in the CBO programme and a regular user of CBO services; and b) two MSM who are not registered in the CBO programme and do not use CBO services. The CRs will ask the seeds to call the identified respondents and obtain consent. Once the respondents agree to participate, the CRs will set up meetings with these respondents and consent will be obtained from the respondents for participation in the assessment. Nine primary seeds will identify 27 respondents in each site. At this level, 27 participants will complete a respondent demographic survey. Each of the 27 respondents will further list out their 30–40 contacts using the same criteria mentioned above. The previous exercise will be repeated with the 27 respondents identifying 3 of their contacts as respondents in the next level. Hence each primary seed will identity 3 respondents and each of the 3 respondents will further identify 3 respondents. That way for each seed, we will interview 12 MSM respondents who are in their network (total of 13 respondents including the seed). In each site with 9 seeds, we will interview 117 MSM respondents in their sexual and social network. In 3 sites, there will be a total of 351 interviews.

**Fig. 2:**
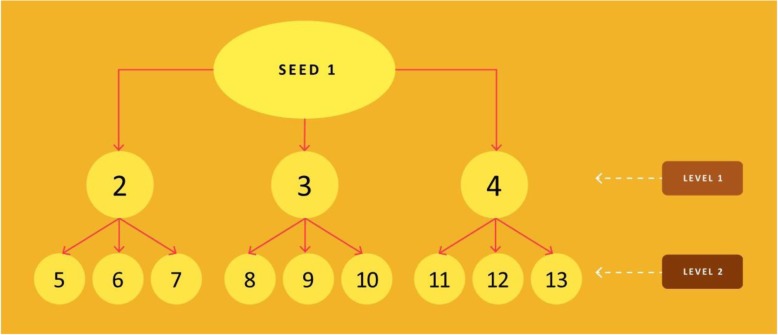
Sample seed-based network

As illustrated in Fig. 2, for each seed, 9 personal demographic forms will be completed along with the network surveys pertaining to the respondents’ sexual partners and friends. For each respondent (including the seed), we expect to get information on approximately 25–30 individuals, based on our past experience. A total of 351 MSM will complete the survey tools/forms, and we will therefore gather information on about 8,500–8,700 MSM in the networks.

A pre-tested questionnaire will be administered face-to-face to collect data for the network study. The data will be collected using paper-based questionnaires and entered by the data entry operators centrally in a database developed in CSPro. Prior to implementing the study, researcher training will also entail a question-by-question discussion and consensus-building process on how to ask each question, based on intent and current terms in common usage. Practice sessions will be conducted to develop the social and sexual networks of respondents.

##### 3. Longitudinal qualitative cohort study

Similar to the network study, the longitudinal study will also directly address secondary outcome 4 and have the same eligibility criteria.

#### Sample recruitment and data collection

The study will provide a sampling frame for the qualitative cohort study. At each of the three sites, the CRs will conduct audio recorded in-depth qualitative interviews with 24 unreached MSM participants: 12 from level 1 and 12 from level 2 (see Fig. 2). Overall, 72 in-depth interviews will be conducted. This qualitative study will facilitate a deeper probe into the social and sexual network that lies beyond the reach of programmes. Over a 12-month period, the CRs, who will encourage these individual to access HIV testing (regular or HIVST) as part of the larger intervention, will follow up with these individuals in person (in a safe location of the participants’ choice) once every four months to discuss experiences around health seeking (especially HIV Self-Testing), stigma and discrimination, and their experiences with meeting up with other MSM for sex and socializing. The community researchers will maintain contact with respondents during the four-month period to ensure that the respondents stay motivated to retain in the study. This follow up information will be captured by employing an open-ended in-depth interview guide and subsequent field notetaking (immediately following the discussions).

##### 4. Routine Programme monitoring

Routine programme monitoring will contribute towards primary outcomes 1–2 and secondary outcomes 4–5. Routine monitoring in the programme will be done using: a) routine reporting tools; and b) polling booth surveys. Routine reporting will include a) Monthly reports from community and facility teams where the intervention team will submit collect data using standard tools and compile them every month and submit b) monthly intervention team meeting where the intervention team will be met every month and a structured questionnaire that will be administered to understand the process of intervention, challenges and facilitators and c) observation of site level activities where project observers/ monitors will attend activities done by outreach team and clinical team to get feedback from MSM on the project activities.

In addition, a population based quantitative assessment will be done using the polling booth survey (PBS) method every 4 months of the intervention. PBS is an unlinked and anonymous group interview process that has been successful in minimizing the social desirability bias that can occur during face-to-face interviews [35]. It is also a low cost and rapid assessment and is normally used to monitor sensitive behaviours, such as condom use. A PBS will be conducted in each of the sites every 3 months to understand progress towards achievement of the objectives and conduct mid-course correction, if necessary. In the PBS, respondents will be randomly selected from hotspots and virtual spaces in groups of 10 individuals. The eligibility criteria for selecting participants for PBS is the same as the eligibility criteria for the intervention. Even though this is a programme monitoring exercise, written consent will be taken from the respondents. The PBS questionnaire will collect information on the coverage and frequency of HIV testing and HIVST, the use of different delivery mechanisms, and linkage to prevention and treatment after testing.

##### 5. Cost effectiveness study

This study will address the secondary outcome 5. Cost data will be collected at the programme level at the three CBOs for all the activities related to different service delivery mechanism. Both financial (representing expenditure on goods and services) and economic (including items for which there is no expenditure, such as donated goods) costs will be included. The latter cost in particular allows for the fact that the HIVST kits are subsidized in Kenya and supplied by the government. Time sheets and other resources such as peer calendars will be used to estimate staff costs related to both direct and secondary distribution of HIVST kits. Data on costs associated with promotion and distribution of HIVST through different channels of delivery will be collected directly.

##### 6. Mathematical modelling

**Fig. 3:**
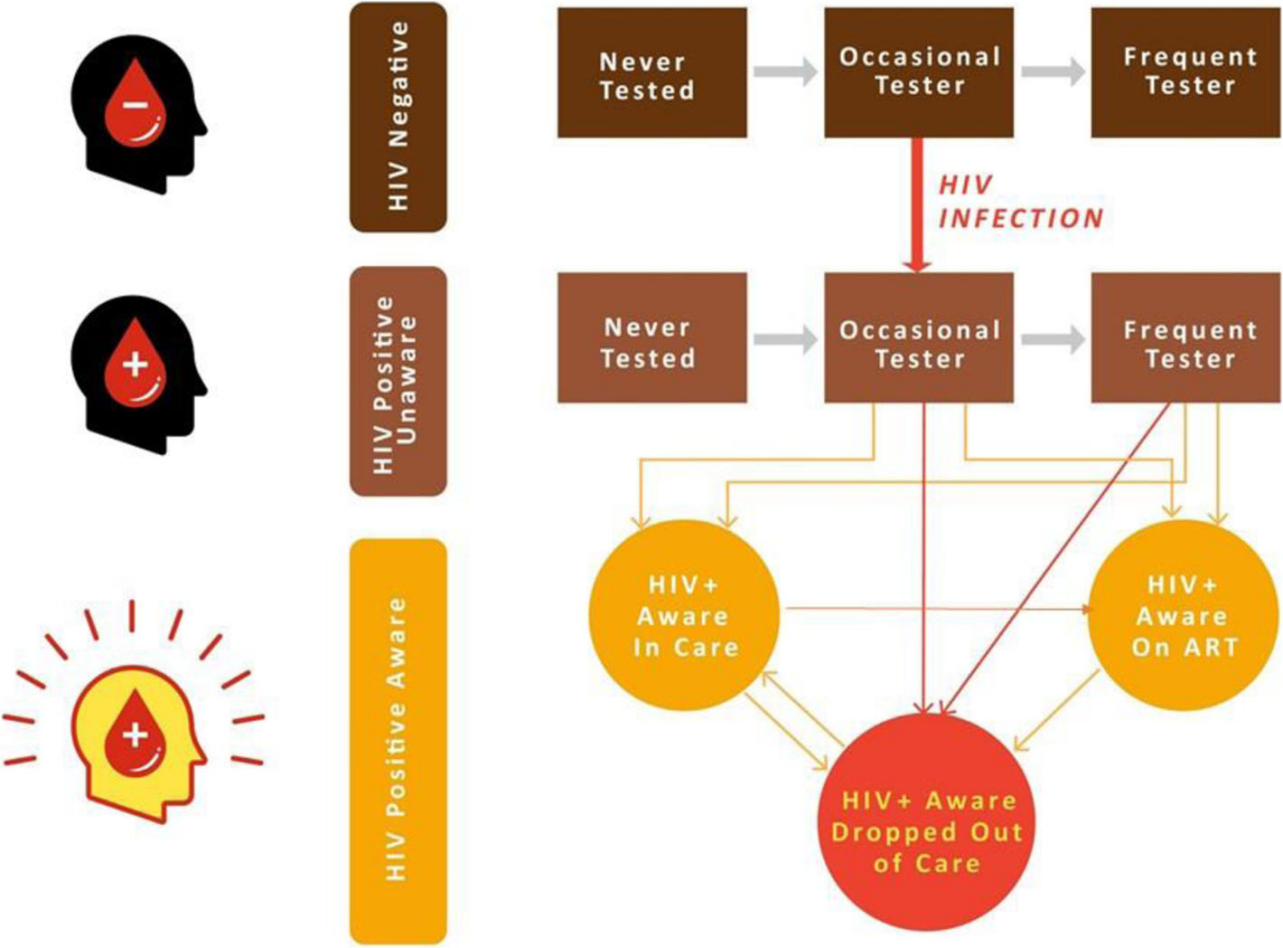
Mathematical model structure

Mathematical modelling will contribute to secondary outcome 6. A deterministic compartmental mathematical model of HIV transmission among MSM in Kenya will be developed that will be tailored to the local epidemiology and informed by findings from the baseline bio-behavioural survey and qualitative study components. The model will also include the general population to capture transmission between MSM and female partners, since a high proportion of MSM in Kenya also have sex with female partners [36]. Figure 3 shows a schematic representation of the structure for HIV testing and treatment in the model. MSM will be divided according to their HIV testing frequency and whether they are HIV negative, HIV positive and unaware of their status, or HIV positive and aware.

The model will be parameterized, calibrated and run for each of the three sites separately. Parameters will come from the county-specific MSM surveys, as well as existing literature. Target-fitting with Latin hypercube sampling, an established method, will be used to calibrate and validate the model. For each calibrated parameter set, simulated counterfactuals will be run. A simulated counterfactual is a run where all parameters are the same as in a calibrated run of the main model, except for those related to the introduction of HIVST. Following the hypothesis of the project, parameters related to the following processes will vary in the simulated counterfactual compared to the main model:

- Coverage and frequency of HIV testing.
- Prevention programme coverage

#### Data analysis

##### 1. **Baseline and Endline bio-Behavioural survey**

Data entry for the baseline and end- line survey will be done by data entry operators centrally in a database developed in CSPro. For the baseline survey, simple frequencies and prevalence will be presented. The descriptive statistics will be calculated using proportions for categorical variables and means (with standard deviations), and medians (with inter-quartile range) for continuous variables. Secondly, a bi-variate analysis of variables will be carried out using chi-square for categorical variables and Student’s t-test for continuous variables. Finally, for the baseline survey, a multivariate analysis of variables associated with HIV testing will be carried out using logistic (or binomial) regression.

At the endline, comparative analysis will be done to answer the study questions. This will be carried out using chi-square and Student’s t-test when comparing the baseline and endline survey, whereas chi-square will be used for linear trends, and simple linear regression will be used after completion of the endline. It will also be possible to perform multivariate analyses of the time trends using multiple logistic (or binomial) regressions, controlling for changes in the socio-demographic characteristics of the study populations over time, if such changes are observed.

##### 2. Socio – sexual network study

An analysis of network study data will be conducted using RDSAT, Net Draw and STATA. RDSAT and Net Draw will be used to understand the size of the MSM network, identify patterns of connection between different sub-populations of MSM, and understand how these connections pivot on characteristics such as age, gender, sexual identity, “outness” or disclosure, program enrollment, and meeting place for sex. Understanding these patterns and the density of connection between different types of MSM at each of the three respective sites (Kisumu, Kiambu and Mombasa) will yield crucial insights into the effective delivery of self-testing technologies during the intervention phase of our project.

##### 3. Longitudinal qualitative cohort study

Qualitative interview transcripts will be analyzed using NVivo 10 data management software. Follow-up qualitative data, in the form of field notes, will be analyzed to further refine the intervention and create tools for more precise outreach planning.

The analysis conducted at baseline and after each round of interviewing at six-monthly intervals will also aid in the review and refinement of the programme strategies. The community intelligence gathered through this process will shed light on barriers and facilitators to HIVST, and how the programme can address them towards making service delivery mechanisms more effective and efficient. We will initiate a process that will allow programme teams to conduct a rapid programmatic secondary analysis of this qualitative data. This additional step will directly benefit program planners by fostering their ownership and utilization of data and findings in their efforts to improve programmes.

##### 4. Routine programme monitoring

For the routine monitoring data, coverage/utilization indicators of program will be analysed over time on monthly basis for each site. Analysis will be done in line with the objectives to illustrate trends in coverage, utilization of HIVST among MSM at county and sub county level. Where applicable, MSM size estimates of 2018 will be used as denominators for intervention outputs. Data will be analysed using STATA 14.0. Qualitative thematic analysis will be done to assess fidelity of intervention, feasibility of intervention and quality of intervention and acceptability of interventions.

3 rounds of polling booth surveys will be done during 12 months of intervention. Data will be collected every 4 months and entered using Microsoft Access and exported to SPSS (v20) and Microsoft Excel for analysis. Descriptive analysis will be performed to produce frequencies, proportions and comparative statistics. Given that PBS data are anonymized and aggregated at the group level (i.e. responses are summarised for each PBS session), the data are unlinked, and do not allow for more complex, multivariate analyses. Data will be weighted using overall and site level selection probabilities, taking into account the sample size and MSM population size estimates, to provide estimates at the overall level.

##### 5. Cost effectiveness study

As we are comparing cost effectiveness of different service delivery methods, the analysis will compare the cost of each activity within the different service delivery mechanism against the primary outcomes of coverage and frequency of use of HIVST. To calculate the cost-effectiveness for each activity, the total costs of the activity will be divided by the outcome achieved by the activity. It is assumed that the start-up costs associated with introducing HIVST into existing programmes are minimal, and thus costs will not be disaggregated into start-up and ongoing costs unless there is evidence to the contrary. Costs will be combined with estimates of impact (both infections averted and disability-adjusted life years averted) from the mathematical modelling to produce estimates of the incremental cost-effectiveness of introducing HIVST as a component of the intervention. This is described below in the mathematical modelling section.

##### 6. Mathematical modelling

The modelling study will use a counterfactual framework described earlier to project impact from the start of self-testing roll-out in 2018 to 2021/2028 (3/10 years). The primary model outputs will be cumulative incident HIV infections over 1, 3 and 10 years. The model will also output the number receiving HIV tests over different time periods, the number diagnosed HIV-positive, and the number receiving ART, disaggregated by whether they used standard HTS or HIVST, as well as DALYs averted (by comparing full and counterfactual models). For each specific analysis, the median and uncertainty bounds (95% credible intervals) will be reported, and an uncertainty analysis will be performed to determine which input parameters were most influential.

#### Data management and storage

All collected data will be entered in a computerized database developed for the study. To ensure quality of data, checks will be done for every 10th interview by the site supervisor and the data manager to verify for completeness and internal consistency. At the end of each field day, the site supervisor will collect all completed interviews and communicate the total number of completed interviews to the national study coordinator. Every week, all completed questionnaires will be couriered to the central project office in Nairobi. Data will be entered centrally and saved at a central secure data server. Double data entry will be conducted for quality assurance. All databases will be password protected and data will be encrypted before transmission over public networks. All completed questionnaires will be kept in a locked cabinet. Questionnaires will not contain identifiable information and will only contain a unique survey code. Access to data will be limited to the data manager, site supervisors, data analysts, and investigators. Results from the HIV rapid-testing will be cross-validated with those from the DBS sample to mitigate any risk from possible low sensitivity of the rapid tests in the field. DBS will be used as the confirmatory test for final results for the study. DBS samples will be shipped within 6 months of collection from the National Laboratory in Nairobi, Kenya to Retrovirology Laboratories in Winnipeg, Canada. The samples will be kept for a period of 5 years and destroyed at the end of that period.

Management of codes from both survey results and HIV test results will be the responsibility of the site supervisors on a daily basis. Continuous quality checks will be performed to ensure that code numbers are recorded properly for each participant. Merging of both data sources (i.e. the laboratory and survey responses) will be conducted under the supervision of the data manager.

### Ethical consideration

Conducting a study with MSM in a country where same sex relationship is criminalized requires careful consideration of the potential benefits and harms that may be caused for those involved in the research. Extensive discussions between members of the CBOs, G10, NASCOP and University of Manitoba were held to identify strategies to protect the safety of participants and researchers, and to try to minimize any social harms resulting from the research. In the context of HIV testing, confidentiality is a key principle. Therefore, training and strict guidelines will be used with the field team to emphasize the importance of confidentiality as a cornerstone of the research. The identity of participants and the information shared by them will not be revealed to anyone who does not work on the study. In addition, unique identifying numbers will be used to identify the questionnaires; no identifying names will be entered with the computer data. Respondents will be informed of the true nature of the study as part of the informed consent process. The CBOs will be also supported to strengthen their crisis management systems within the organisations such that any situation challenging situation that may arise can be sensitively managed. NASCOP also has protocols on research including patient safety that will be adhered to.

The study has obtained approval from the institutional review boards of the Kenyatta National Hospital - University of Nairobi, Kenya (P557/08/2018) and the University of Manitoba – Health Research Ethics Board, Canada (HS22205).

The results of this study will be disseminated through the Key Population Technical Working Group and the HIV Testing Services Technical Working Group led by NASCOP at both national and county level at regular intervals, as each step of the study is completed. The findings will be also shared with G10 members who are part of the MSM CBOs representing different regions of Kenya. Community meetings will be organized at the study sites to share the findings with MSM community members. Study results will also be disseminated through peer reviewed journals, presentations at conferences, stakeholders (global and local), and with policy makers in Kenya including the Kenyan Ministry of Health. A study closure report will be submitted to the institutional review boards in Kenya and Manitoba.

## Discussion

As far as we know, no study has adopted a programme science framework to explore the effectiveness of HIVST within a scaled up HIV prevention intervention among MSM in reducing the undiagnosed proportion of MSM and time to linkage to post-test services in Africa. This study uses programme science principles [26] and aims to answer questions that are emerging from programmes, and use the answers generated through the study in programme practice and policy. The study will be embedded within a scaled-up HIV prevention programme for MSM in the three counties. The uniqueness of the study is that it uses a community-based research approach and acknowledges the views of MSM community members and community led organizations on one side, and the Ministry of Health on the other side, as integral partners in the inquiry process. Community-based research practices share decision-making and co-ownership of the research products among partners, co-learning and reciprocal transfer of knowledge in a self-reflective manner, strengthening of research and programme development capacity, and relevance to locally identified needs [37, 38]. The development of this protocol has been through a participatory process where all partners have contributed equally to defining the research questions and designing the research itself. The CBO partners will be involved in using the findings in designing interventions and implementing the intervention. Partnership with the National Key Population Programme through the Ministry of Health will ensure that the research is based on the needs of the programme, and research findings will be used to develop policy and scale up existing programmes.

As most data about the use of HIVST will be self-reported by MSM, their partners, and sexual networks, bias may be a limitation of the study. Participation in the study may reveal that MSM are engaging in illegal and stigmatized behaviors, particularly same sex relationships, and may subject persons to discrimination and potential harm. HIV serostatus may also subject participants to stigma and discrimination if inadvertently revealed to persons outside the study. The study plans to develop a risk mitigation strategy in partnership with the CBOs and the national and county government to address these challenges. Sensitization will be done with various stakeholders in the counties, and the county Ministry of Health officials will be actively involved. The crisis management system within CBOs will be strengthened to ensure that support can be provided immediately in case of a crisis arising from the study.

In conclusion, this study will provide additional evidence on the impact of integrating HIVST interventions within already existing HIV prevention and treatment programmes for MSM. This study will provide an important addition to the limited evidence on HIVST and MSM in Africa. A unique feature of the study is the partnership between an academic institution, MSM led community-based organizations, and government institutions, in designing, implementing and applying the findings of the research for policy and practice.

## Data Availability

The datasets used and analysed during the study will be available from the corresponding author on reasonable request.

## Abbreviations

AIDS: Acquired Immune Deficiency Syndrome
CBO: Community-Based Organization
CR: Community Researchers
CSPro: The Census and Survey Processing System
DBS: Dried Blood Spot
DIC: Drop-In Centre
FSW: Female Sex Worker
HAPA Kenya: The HIV & AIDS People’s Alliance of Kenya
HIV: Human Immuno-Deficiency Virus
HIVST: HIV Self-Test
HTS: HIV Testing Services
ID: Identification
KP: Key Population
MAAYGO: Men Against AIDS Youth Group
MPEG: Mamboleo Peer Empowerment Group
MSM: Men Who Have Sex With Men
MSW: Male Sex Worker
NASCOP: National AIDS and STI Control Programme
PBS: Polling Booth Survey
PWID: People Who Inject Drugs
RDSAT: The Respondent Driven Sampling Analysis Tool
STATA: Software for Statistics and Data Science
STI: Sexually Transmitted Infection
